# Brain structure changes associated with depression outcome in adolescents bullied throughout adolescence

**DOI:** 10.1192/j.eurpsy.2023.293

**Published:** 2023-07-19

**Authors:** M.-L. Paillere-Martinot, A. Briffod, P.-A. Beaudoin, O. Hassani, J.-L. Martinot, E. Artiges

**Affiliations:** 1 INSERM U1299 “Developmental trajectories & psychiatry”, Université Paris-Saclay, Ecole Normale Supérieure Paris-Saclay - CNRS - Centre Borelli; 2 Ecole CentraleSupélec, Université Paris-Saclay, CentraleSupélec, Gif-sur-Yvette, France

## Abstract

**Introduction:**

Being bullied in adolescence has been associated with developing depressive symptoms in adulthood.

**Objectives:**

We sought to describe the trajectories of peer victimization across adolescence and their relationships with grey matter volumes and depression outcomes in young adulthood.

**Methods:**

Community adolescents from the IMAGEN database (n = 724) with both peer victimization and neuroimaging data were included. A longitudinal clusterization method (normal mixture model) was used to analyze the bullying scores at baseline (age 14), and at follow-ups at age 16, 18 and 22. Relations between clusters and brain volumes or depression diagnosis were examined using logistic and linear multivariate regression models.

**Results:**

Three victimization trajectories were observed. A first trajectory included participants who were never bullied and had no depression outcome, a second trajectory identified participants who were bullied at age 14 and 16 only, and had no depression outcome, and finally, a third trajectory of continuous bullying throughout adolescence to young adulthood (age 22) that was significantly associated with depression outcomes (r=0.87, p=0.0004). In addition, the continuously bullied participants displayed larger volumes of bilateral hippocampus, posterior cingulate cortex and right putamen at age 22.

**Conclusions:**

These data confirm that chronic peer victimization throughout adolescence is associated with brain structure changes and might increase vulnerability to depressive disorders. They highlight the need for preventive school interventions in early adolescents.

**Disclosure of Interest:**

None Declared

